# Understanding the influence of plant genetic factors on rhizosphere microbiome assembly in *Panax notoginseng*

**DOI:** 10.3389/fmicb.2024.1479580

**Published:** 2024-12-16

**Authors:** Liping Shi, Mingming Yang, Guangfei Wei, Xiuye Wei, Fei Hong, Jiaxiang Ma, Zhe Wu, Yuqing Zheng, Miyi Yang, Shilin Chen, Guozhuang Zhang, Linlin Dong

**Affiliations:** ^1^State Key Laboratory for Quality Ensurance and Sustainable Use of Dao-di Herbs, Institute of Chinese Materia Medica, China Academy of Chinese Medical Sciences, Beijing, China; ^2^Institute of Herbgenomics, Chengdu University of Traditional Chinese Medicine, Chengdu, Sichuan, China; ^3^Zhangzhou Pien Tze Huang Pharmaceutical Co., Ltd., Zhangzhou, China

**Keywords:** rhizosphere soil, functional microbiomes, biocontrol bacteria, fungal plant pathogens, community succession, plant genetic network

## Abstract

**Introduction:**

Functional rhizosphere microbiomes (FRM) are critical for plant health and yield. However, the ecological succession of FRM and their links to plant genetic factors across the life cycle of perennial plants remain poorly understood.

**Methods:**

This study profiled FRM, including plant-beneficial bacteria (PBB) and fungal plant pathogens (FPP), across different developmental stages of *Panax notoginseng*.

**Results:**

The biodiversity of both PBB and FPP were significantly higher in rhizosphere compared with farmland soil, and exhibited different succession patterns with plant growth. The relative abundance of PBB, but not FPP, decreased after plant cultivation. There were significantly negative correlations between FPP and PBB, particularly the biocontrol subgroup (*ρ* = −0.56, *p* < 0.001). The antagonistic effects of biocontrol bacteria against fungal pathogens were further validated by *in vitro* assays. The fitting of neutral community model indicated that the deterministic assembly of PBB, especially the biocontrol subgroup, was the strongest at the 3rd-year root growth stage of *P. notoginseng*. Plant genes involved in protein export, biosynthesis of alkaloids and amino acids were identified as drivers of the deterministic assembly of biocontrol subcommunity by RNA-Seq analysis. Additionally, a total of 13 transcription factors potentially regulating the expression of these biosynthesis genes were identified through co-expression network. In summary, this study unveils the succession patterns of FRM throughout the life cycle of *P. notoginseng* and the underlying plant genetic mechanisms, providing valuable insights for developing new plant disease management strategies by manipulating microbes.

## 1 Introduction

The rhizosphere, the soil region closely surrounding plant roots, is a unique habitat teeming with a diverse array of microbes, both beneficial and harmful to the host plant (Tan et al., 2017). These complex microbial communities and their interactions, such as antagonism and mutualism, significantly impact plant development, health, and yield (Bulgarelli et al., 2012; Etesami and Glick, 2020; Li et al., 2021). In recent years, plant-beneficial bacteria (PBB)^1^ in the rhizosphere have garnered increasing attention due to their potential role in sustainable agriculture (Haskett et al., 2021; Nerva et al., 2022). Functionally, PBB can be categorized into various subgroups, including biocontrol taxa (Bioc)^2^ that antagonize pathogens, plant-growth-promoting taxa (PGP)^3^ that enhance nutrient absorption in host plants, and stress resistance provision taxa (SR)^4^ capable of alleviating plant abiotic stress (Banerjee and van der Heijden, 2023; Li et al., 2023). Extensive studies on the taxonomy, functions, and geographic distribution of PBB have improved our understanding of their functional mechanisms and global community structures (Haskett et al., 2021; Li et al., 2023). However, the assembly mechanisms of PBB in the rhizosphere and their succession across different developmental stages of the host plant, particularly perennial species, remain elusive. This understanding is crucial for effectively utilizing PBB to enhance plant performance in complex environments and achieving sustainable agriculture (Cordovez et al., 2019). Conversely, soil-borne fungal pathogens are the primary cause of plant diseases and yield reduction (Peng et al., 2023). For many plant species, especially under continuous monoculture, there is a tendency for the accumulation of specialized fungal plant pathogens (FPP)^5^ in the rhizosphere, leading to increased disease incidence (Fones et al., 2020). Thus, understanding the assembly and succession of these harmful taxa is also vital for developing targeted and environment-friendly control strategies to maintain plant health (Bai et al., 2022).

During plant development, the succession of rhizosphere functional microbiomes, such as PBB and FPP, is influenced by a combination of host plant genetic factors, biotic interactions, environmental conditions, and stochastic forces (Cordovez et al., 2019). For example, plants can contribute to microbiome succession by altering edaphic properties and soil nutrient availability (Hannula et al., 2021; Xun et al., 2024). Antagonistic interactions between Bioc taxa and pathogens exemplify the biotic interplays affecting microbial community assembly (Perez-Gutierrez et al., 2013). Additionally, continuous dispersion, birth, and death of microbes are significant drivers of microbial dynamics, even in plant-associated habitats (Zhang et al., 2022). In summary, functional microbes can colonize the rhizosphere soil through dispersal from soil, air, and plants, forming a specific and dynamic community under the integrative effects of host and biotic factors, environmental conditions, and ecological stochasticity (Berendsen et al., 2018; Li et al., 2023; Song et al., 2021). Although the extreme complexity of influencing factors presents a major challenge in studying rhizosphere functional microbiome succession, research from an ecological assembly perspective can provide valuable insights into the interplay between plant development and the functional microbiome (Cordovez et al., 2019). From a metacommunity viewpoint, microbial community assembly results from both deterministic and stochastic processes, with the relative importance of these processes varying across space and time (Zhang et al., 2021). Deterministic processes include ecological selection imposed by biotic interactions and abiotic factors, such as environmental filtering, while stochastic processes encompass dispersal events, random birth-death events, and ecological drifts (Jiao et al., 2020; Xie et al., 2022). These ecological forces collectively shape the succession patterns of rhizosphere functional groups (e.g., PBB, FPP) during plant development. Various mathematical models, including neutral theory-based neutral models and null-model based NST (Ning et al., 2019; Sloan et al., 2006), have been developed to assess the relative contribution of different ecological processes to microbial community assembly. These models, widely used in diverse ecosystems, including plant-associated habitats, have provided critical insights into the assembly and succession mechanisms of ecologically important microbial communities (Zhou and Ning, 2017).

Another challenge in functional microbiome studies is identifying microbial members that are beneficial or harmful to the host plant. Traditional approaches rely on culturing and isolating microbes, followed by *in vivo* and *in vitro* validation experiments. However, due to technical limitations, not all microorganisms inhabiting plant-associated niches can be cultured and isolated (Compant et al., 2019). Sampling only local communities, such as limited rhizosphere soil samples, often fails to represent the entire species pool of the corresponding niche (Leibold et al., 2004). Additionally, some microbial functions related to plant phenotypes are only expressed in specific environments, such as with opportunistic plant pathogens (Brown et al., 2012). Recently, significant efforts have been made to assign functional characteristics to microbial taxa. For instance, the PBB database includes soil bacteria with experimentally verified plant-beneficial functions across three mechanisms: biocontrol, growth promotion, and stress resistance (Li et al., 2023). In the realm of fungi, Põlme et al. (2021) introduced the FungalTraits database, which amalgamates pre-existing databases and expert knowledge. These resources provide promising avenues for deciphering functional microbiomes.

*Panax notoginseng* (Burk.) F. H. Chen is a renowned and valuable perennial medicinal plant, highly esteemed for its medicinal and economic value (Zhang et al., 2021). Typically, *P. notoginseng* is cultivated for a minimum of 3 years before harvesting, with annual vegetative, flowering, and root inflation stages (Wang et al., 2016). Our previous studies have shown that the rhizosphere microbial community of *P. notoginseng* undergoes significant changes during its growth, particularly in the third year of root inflation growth, where the variations of microbial community were active (Wei et al., 2022). The variations in functional rhizosphere microbiomes during plant growth might contribute to the soil-borne disease occurrence. For example, *Fusarium oxysporum*-induced root rot was often attributed to the disruption of soil microbiome homeostasis (Li et al., 2022). The introduction of beneficial microbial communities was expected to recover the imbalance of micro-ecology to a state of relative health, thus reducing the abundance of plant pathogens and plant mortality (Zhang et al., 2020). However, the succession of the functional rhizosphere microbial community during growth stages is still unclear. Understanding the assembly mechanisms and succession patterns of rhizosphere functional groups alongside *P. notoginseng*’s development can offer insights into the dynamics of the rhizosphere microbiome in relation to plant health and lay the groundwork for developing microbe-based agricultural management strategies. In this study, we identified the rhizosphere functional microbial communities of *P. notoginseng* at different developmental stages with the goals of: (i) revealing the succession patterns of distinct functional groups, including PBB and FPP, and their potential interactions; (ii) quantifying the relative contribution of various ecological processes to the assembly of functional microbiomes; and (iii) linking the succession of beneficial groups to root transcriptional regulation network throughout the development of *P. notoginseng*. Considering the functional potential of PBB and the biological characteristics of *P. notoginseng*, we hypothesize that: (i) the relative abundance of PBB, especially Bioc taxa, shows negative correlations with that of FPP, indicating potential antagonistic effects; (ii) the 3-year root inflation stage of *P. notoginseng* exerts strong selection on functional rhizosphere microbiomes; (iii) biosynthesis-related plant functions drive the succession of functional microbial communities.

## 2 Materials and methods

### 2.1 Collection of rhizosphere soil of *P. notoginseng* across different developmental stages

The quality of *P. notoginseng* root was strongly affected by cultivation area, with Wenshan Prefecture, Yunnan Province being the core production area of high-quality medicinal materials. For a better description of general patterns of rhizosphere functional microbiomes, the present study collected rhizosphere soil of *P. notoginseng* in five fields of three sites across the Wenshan Prefecture, including Pingba (23°14′29.5” N, 104°5′3.0″ E; PBA, PBB, and PBC), Yanshan (23°34′56.22” N, 104°19′49.05″ E; YS), and Qiubei (23°49′46.99″ N, 104°06′12.99″ E; QB). As described in the previous study (Wei et al., 2022), soil samples were collected during a 2-year growth from early 2016 to the end of 2017. In brief, 1-year-old seedlings were transplanted into each field and then cultivated according to Good Agricultural Practices (GAP) (Zhang et al., 2021). In each field and each sampling, three separated plots (1.4 m^2^ × 8.0 m^2^) were randomly selected as biological replicates, from each the rhizosphere soil was collected from 10 individual plants following with mixing. Specifically, 10 randomly selected healthy plants in each plot were gently dug out using a sterilized shovel and represented one sample, and bulk soil was removed by shaking the plants (Ahmed et al., 2022). Rhizosphere soil sample was collected through gentle shaking and squeezing in a sterile ziplock bag (Zhang et al., 2023). Before transplanting, bulk soil (BL) was also collected from 10 randomly selected locations in each plot. Rhizosphere soil samples were collected from three developmental stages of *P. notoginseng* each year, including vegetative growth stage (2-year and 3-year vegetative, 2YV and 3YV), flowering stage (2YF and 3YF), and root inflation stage (2YR and 3YR). A total of 105 soil samples were obtained (seven stages * 5 fields * 3 plots). After sampling, soil was sieved (2 mm) and stored at −80°C for further analysis.

### 2.2 Soil DNA extraction and amplicon sequencing

Total DNA was extracted from soil samples using the FastDNA SPIN Kit for soil (MoBio Laboratories, Inc., United States), and extracted DNA was stored at −80°C for future use (Zhang et al., 2021). The V4-V5 region of bacterial 16S rRNA gene was amplified using 515F (5′-GTGCCAGCMGCCGCGG-3′) and 907R (5′- CCGTCAATTCMTTTRAGTTT-3′) primer pair. Fungal ITS region was amplified using ITS1F (5′-CTTGGTCATTTAGAGGAAGTAA-3′) and ITS2R (5′- GCTGCGTTCTTCATCGATGC-3′) primer pair (Wei et al., 2022). Sequencing was performed on Illumina MiSeq PE 250 platform (Shanghai Biozeron Co., Ltd., China). Raw FASTQ files were demultiplexed, quality filtered, and denoised to generate amplicon sequence variants (ASVs) using the QIIME2-DADA2 pipeline (Bolyen et al., 2019; Callahan et al., 2016). The representative sequences of ASVs were taxonomically classified using the machine-learning-based classification methods implemented in QIIME2. Bayes-trained classifiers based on SILVA (v138) and UNITE (v8.0) were used to assign bacterial and fungal ASVs, respectively (Nilsson et al., 2019; Pruesse et al., 2007). Bacterial ASVs assigned to plant chloroplast and mitochondria were removed to eliminate host contamination. Low-abundance ASVs (total reads <10) were also removed to reduce the impact of potential sequencing error on results (Jiao and Lu, 2020). Bacterial and fungal feature tables were rarefied to an even library size of 2,339 and 3,827 reads, respectively. A total of 5,938 and 1,245 bacterial and fungal ASVs were obtained. The raw sequence data supporting amplicon analysis could be downloaded from the NCBI Sequence Read Archive (SRA) under the Bioproject number PRJNA559079.

### 2.3 RNA-seq and transcript assembly

Roots of 2-year and 3-year-old *P. notoginseng* at different developmental stages corresponding to rhizosphere soil samples were collected from Wenshan Prefecture (Wei et al., 2020). The entire root was dug out, washed, and ground into powder using liquid nitrogen. Total plant RNA was extracted from bulk root using plant RNA Isolation Mini Kit (BioTeke, Beijing, China). The RNA-Seq library was prepared according to the Illumina instruction. Sequencing was performed on Illumina HiSeq platform. After quality filtered, the FASTQ data was aligned to reference genome of *P. notoginseng* using HiSAT2 (Jiang et al., 2021; Kim et al., 2019). The abundance of transcripts was counted using featureCounts (Liao et al., 2014). The transcript abundance was normalized as fragments per kilobase per transcript per million mapped reads (FPKM values). Raw sequencing data could be downloaded from NCBI (PRJNA488357) and NGDC (PRJCA024789).

### 2.4 Isolation of FPP and rhizosphere biocontrol bacteria as well as antagonism test

The fungal pathogen *Fusarium oxysporum* has been reported as the major pathogen associated with the root rot of *P. notoginseng* (Ma et al., 2013). The *F. oxysporum* strain was isolated from *P. notoginseng* plant suffered from root rot in Yunnan Province and was used as the target for antagonism test. Rhizosphere bacteria isolates were obtained by inoculating soil suspension on 1/10 TSA plates (Zhou et al., 2022). The obtained bacterial strains were identified by reconstructing Neighbor-Joining phylogenetic tree based on 16S rDNA sequences. Antagonism test was conducted to estimate the inhibitory ability against *F. oxysporum* of potential biocontrol bacteria (Zhou et al., 2022).

### 2.5 Statistical analysis

All statistical analyses were conducted in R environment (v4.2.2).^6^ R codes used for statistical analyses are available at: https://github.com/githubzgz.

PBB were identified based on the PBB database (Li et al., 2023). Briefly, bacterial ASVs belonging to genus with beneficial functions, including biocontrol, stress resistance, and plant-growth-promoting capabilities, were identified as PBB taxa. Potential fungal plant pathogens were identified on the basis of “Plant pathogenic capacity” term in the FungalTraits database (Põlme et al., 2021). Alpha diversity indices of functional microbiomes, including richness, Chao1, and Shannon, were calculated using the vegan package (Dixon, 2003). The distribution of microbial abundance data is skewed, which does not meet the conditions of normal distribution. Kruskal-Wallis test, Nemenyi test, and nonparametric ANOVA (PERMANOVA) do not rely on the assumption of data distribution (Weiss et al., 2017). Therefore, Kruskal-Wallis test was used to evaluate the *α*-diversity differences among distinct growth stages, followed by the Nemenyi test for multiple comparisons using the PMCMRplus package (Pohlert, 2021). PERMANOVA was used to assess the explanatory power of developmental stages and sampling sites on microbial community variations represented by Bray-Curtis dissimilarities. This analysis was conducted using the adonis2 function in vegan package (Dixon, 2003). *β*-dispersal of microbial communities at different growth stages were calculated using the vegan package. Contributions of ASVs to the overall β-diversities were calculated using the simper function in vegan package. The abundance successions of the top 15 ASVs that were most important to community variations were fitted using local polynomial regression. Community variations were visualized by principal coordinates analysis using the vegan package.

Spearman correlations were calculated between the richness and relative abundance of PBB and FPP, aiming to explore the potential antagonism between functional groups. For each developmental stage, the spearman correlations between the relative abundance of FPP and widespread ASVs (occurring in more than half of samples) were also calculated and mapped on the phylogenetic tree constructed by FastTree. The phylogeny was visualized using the ggtree package (Yu et al., 2016).

To evaluate the relative importance of stochastic and deterministic processes in community assembly of rhizosphere functional microbiomes, we applied the Sloan’s neutral model (Sloan et al., 2006) and the null model-based NST method (Ning et al., 2019). In Sloan’s neutral model, the neutral taxa ratio (NTR) was calculated to represent the relative importance of neutral process to community assembly (Ji et al., 2020; Sloan et al., 2006). Normalized stochasticity ratio (NST) was used to quantify the contribution of ecological stochasticity to the assembly of functional microbial communities. NST analysis was performed using the NST package (Ning et al., 2019).

For linking root transcriptome profiles to the succession of community assembly of rhizosphere Bioc taxa, the transcripts were grouped into different clusters using fuzzy c-means algorithm with the Mfuzz package (Kumar and Futschik, 2007). Clusters exhibiting similar trends with the deterministic assembly of Bioc taxa were further analysed. Enrichment analysis of genes in different clusters was conducted using the clusterProfiler package (Wu et al., 2021). For cluster2 with expression trend similar to the deterministic assembly of Bioc taxa, a gene co-expression network was constructed using spearman correlation analysis (Spearman *ρ* > 0.9; BH adjusted *p* < 0.001). Genes belonging to the enriched biosynthesis pathways were extracted, and a subnetwork comprising these genes and connected genes was visualized using the Cytoscape platform (Shannon et al., 2003). Direct connections between transcription factors (TF) and biosynthesis-related genes were also extracted and visualized.

## 3 Results

### 3.1 Identification of functional microbial communities in the rhizosphere of *P. notoginseng*

**Figure 1:**
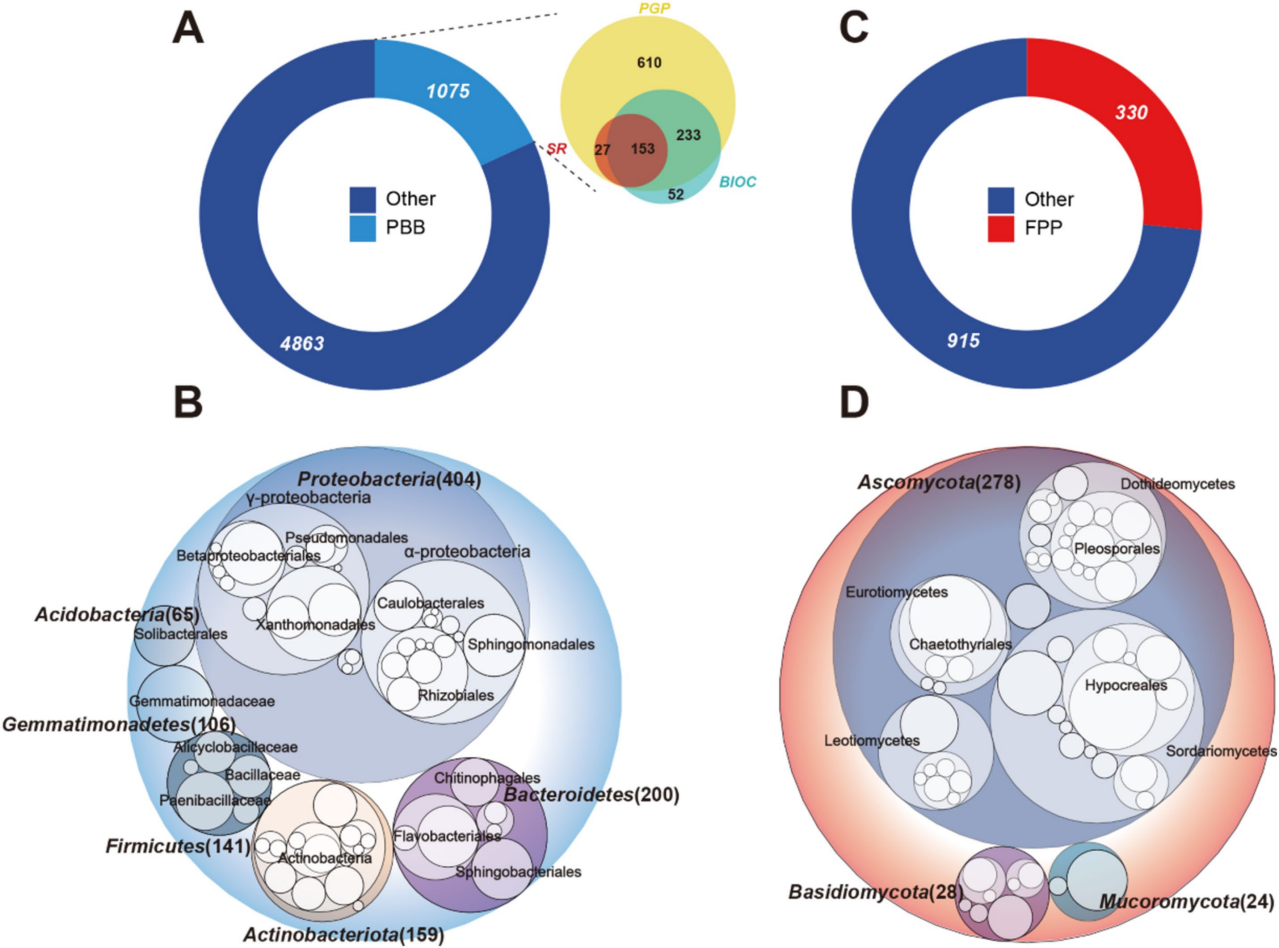
Potential PBB and FPP in the rhizosphere of *P. notoginseng*. Donut plots displayed the number of ASVs in PBB **(A)**, FPP **(C)**, and provided information on the number of subclasses outside the circular plot. Numbers represented the number of ASVs. Circular packing showed PBB ASVs **(B)** and FPP ASVs **(D)** at the phylum level, the color of the circle represented different phylum, with the diameter of the circle being proportional to the number of ASVs. PBB, plant-beneficial bacteria; FPP, fungal plant pathogens; PGP, plant-growth-promoting taxa; BIOC, biocontrol taxa; SR, stress resistance provision taxa.

In the primary cultivation area of *P. notoginseng* in Yunnan Province, a total of 15 bulk soil and 90 rhizosphere soil samples were collected at various plant growth stages. These stages included farmland (BL), two-year and three-year vegetation (2YV and 3YV), flowering (2YF and 3YF), and root inflation (2YR and 3YR). Following quality control measures, chimera filtering, and low-abundance filtering, we successfully identified 5,938 bacterial and 1,245 fungal amplicon sequence variants (ASVs). From these, based on the PBB database, 1,075 bacterial ASVs were identified as PBB. These comprised 1,023 ASVs associated with plant-growth-promoting characteristics, 438 ASVs related to biocontrol characteristics, and 180 ASVs linked to stress resistance traits. A total of 153 bacterial taxa demonstrated all three plant-beneficial characteristics (Figure 1A). At the phylum level, the PBB communities were primarily composed of Proteobacteria, which represented 37.6% of the total PBB ASV count, followed by Bacteroidetes, Actinobacteria, Firmicutes, Gemmatimonadetes, and Acidobacteria (Figure 1B). Additionally, we identified 330 FPP ASVs in the rhizosphere soil, which accounted for approximately 27% of the total fungal ASV count (Figure 1C). The potential FPP communities were predominantly Ascomycota, constituting 84.2% of the total FPP ASV count, with Basidiomycota and Macoromycota following (Figure 1D). These findings indicate that the rhizosphere of host plants is rich in both PBB and FPP.

### 3.2 PBB and FPP showed different succession patterns in terms of biodiversity and community composition

**Figure 2:**
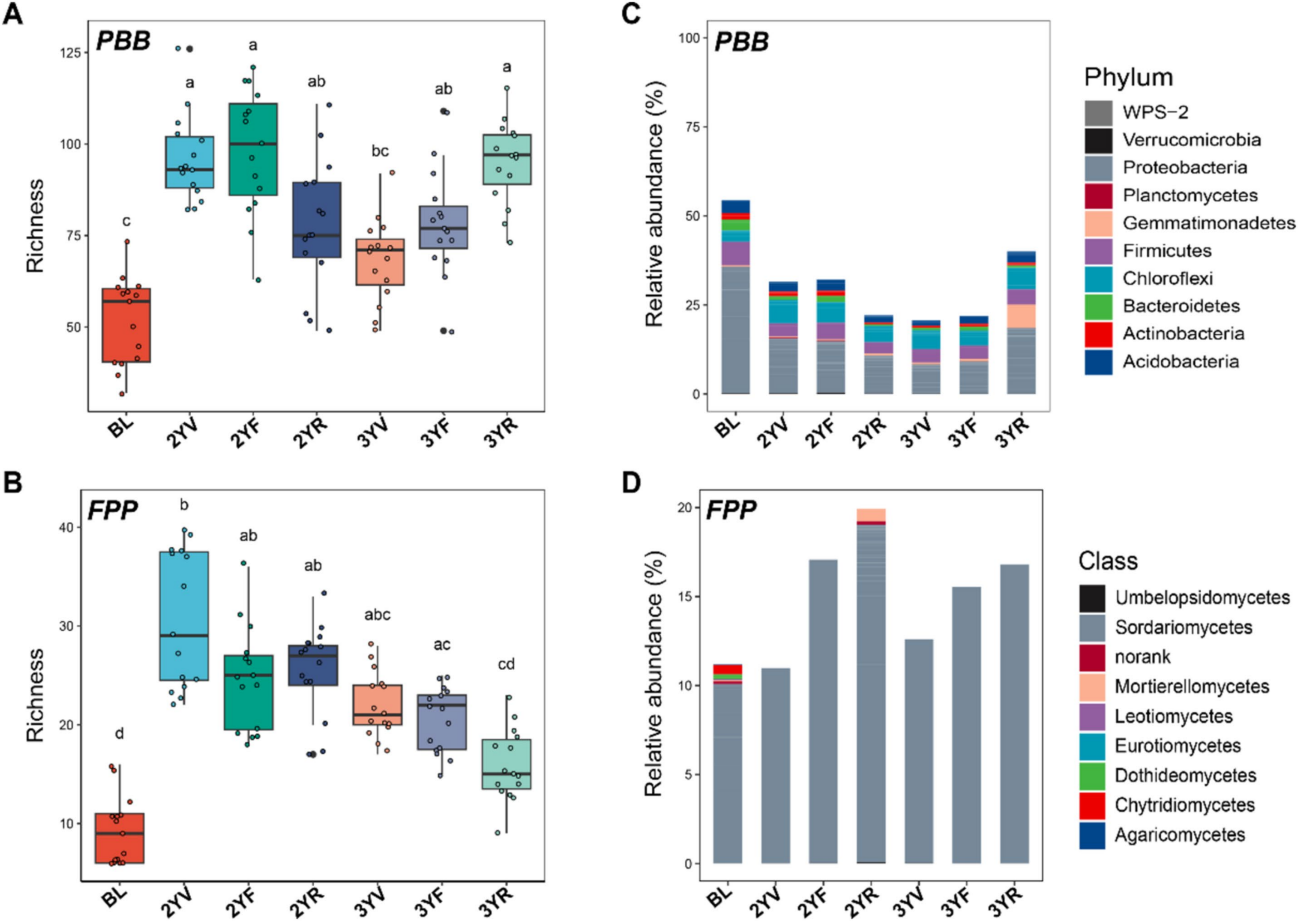
Changes in *α*-diversities and composition of PBB and FPP communities with *P. notoginseng* growth. PBB **(A)** and FPP **(B)** α-diversities at different growth stages were represented by ASV richness index. Different letters above the boxes indicate a significant difference determined by Kruskal-Wallis followed by Nemenyi test (*p* < 0.05). Shifts in the compositions of PBB **(C)** and FPP **(D)** communities with *P. notoginseng* growth. BL, 2YV, 2YF, and 2YR represent bulk soils and the 2-year vegetative, flowering, and root growth stages, respectively. 3YV, 3YF, and 3YR denote the 3-year vegetative, flowering, and root growth stages, respectively. PBB, plant-beneficial bacteria; FPP, fungal plant pathogens.

The communities of potential PBB and FPP exhibited significant shifts across the developmental stages of *P. notoginseng*, both in terms of *α*-diversities (Figures 2A,B) and community compositions (Figures 2C,D). The α-diversity of PBB in the rhizosphere soil was notably higher than in bulk land (BL) soil (Kruskal-Wallis followed by Nemenyi test, *p* < 0.05) (Figure 2A; Supplementary Figure S1). Within the rhizosphere, PBB biodiversity decreased from the 2YV to the 3YV stages, then significantly increased, reaching a peak at the 3YR stage. The dynamic succession pattern of α-diversities was similar for both PBB and its subgroup communities (Supplementary Figure S2). However, the relative abundance of PBB was lower in the rhizosphere soil compared to BL (Figure 2C; Supplementary Figure S2). The trend in PBB relative abundance from 2YV to 3YR mirrored that of the PBB α-diversity, with a marked increase observed from the 3YF to 3YR stages. Regarding community composition, the relative abundance of Proteobacteria and Firmicutes in PBB was substantially lower in the rhizosphere soil compared to BL (Figure 2C). At the 3YR harvest stage, the relative abundance of Proteobacteria and Gemmatimonadetes increased significantly compared to other growth stages (Figure 2C).

Similarly, the α-diversity of FPP in the *P. notoginseng* rhizosphere was significantly higher than in BL (Figure 2B). After planting, FPP biodiversity in the rhizosphere gradually decreased from 2YV to 3YR (Figure 2B). The pattern of FPP relative abundance was consistent between the two-year and three-year stages, with an increase in relative abundance from the vegetation to root inflation stages each year (Figure 2D). Sordariomycetes was the dominant fungal class throughout the sampling period (Figure 2D). These findings underscore the distinct succession patterns of rhizosphere functional microbiomes across *P. notoginseng*’s developmental stages, highlighting the divergence in biodiversity and community composition between PBB and FPP.

### 3.3 Temporal dynamics of PBB and FPP community structures across *P. notoginseng* developmental stages

**Figure 3:**
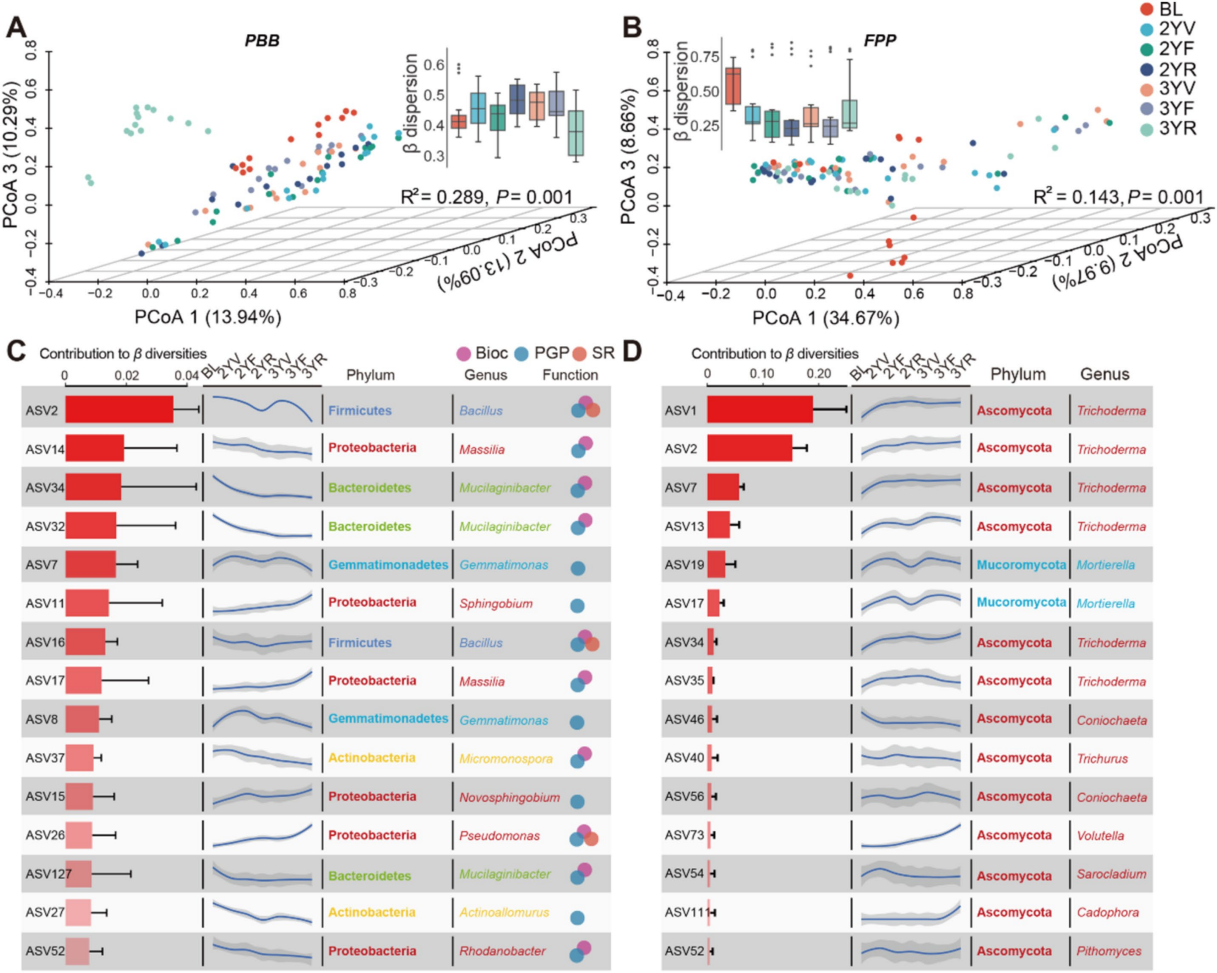
Changes in community structures of PBB and FPP across developmental stages of *P. notoginseng*. Principal coordinates analysis (PCoA) based on Bray-Curtis distances displaying significant differences among distinct sampling stages of PBB (R^2^ = 0.289, *p* = 0.001, PERMANOVA) **(A)** and FPP (R^2^ = 0.143, *p* = 0.001, PERMANOVA). **(B)** The inside boxplots represented *β* dispersion of two functional communities, respectively. The top 15 bacterial **(C)** and fungal **(D)** ASVs that were important to the community variations. Bar plots indicated the contributions of ASVs to β-diversities; curve graphs were obtained by fitting the relative abundance of ASVs to developmental stages. Dots on the right of **(C)** represented different functional subgroups in PBB. PBB, plant-beneficial bacteria; FPP, fungal plant pathogen.

PERMANOVA revealed that the developmental stages significantly drove the community structure variations in PBB (R^2^ = 0.289, *p* = 0.001) and FPP (R^2^ = 0.143, *p* = 0.001) as measured by Bray-Curtis dissimilarities (Figures 3A,B). A distinct separation among PBB collected from different stages was showed by PCoA, and the 3YR samples could be separated from others (Figure 3A). PCoA of FPP also showed significant separation between BL and rhizosphere soil samples (Figure 3B). To quantify the community variations within each developmental stage, the *β*dispersion represented by the mean distances to group centroid were calculated and compared (Figures 3A,B). Although no statistically significant differences exhibited among different developmental stages, the β dispersion of both PBB and FPP showed apparent succession patterns. For PBB, the β dispersion firstly increased from BL to 2YV, then decreased from 3YF to 3YR (Figure 3A). In contrast to PBB, the β dispersion of FPP showed a trend of first decreasing and then increasing along the developmental stage (Figure 3B). These results revealed that the community structure variations of PBB and FPP communities were active at the 3YR stage.

To identify ASVs important for community variations, the contributions of ASVs to β diversities were calculated and the succession patterns of top 15 bacterial and fungal ASVs were fitted (Figures 3C,D). The relative abundance of 8 PBB ASVs showed decreasing tendency with plant growth, including members of *Bacillus*, *Mucilaginibacter*, *Actinoallomurus*, and *Rhodanobacter*. While the relative abundance of ASVs belonging to *Pseudomonas*, *Novosphingobium* and *Sphingobium* increased from BL to 3YR stage (Figure 3C). Most of the top 15 FPP ASVs showed an uprush from BL to 2YV, following with a stable abundance from 2YV to 3YR, mainly including members of *Trichoderma* (Figure 3D). However, the relative abundance of fungal ASV73 (*Volutella*) exhibited continuous growth after planting, and the uprush of fungal ASV11 (*Cadophora*) occurred from 3YF to 3YR (Figure 3D). These results identified different succession patterns of functional taxa that were critical for microbiome variations.

### 3.4 Rhizosphere biocontrol bacteria exhibited the strongest antagonistic interactions with FPP during *P. notoginseng* growth

**Figure 4:**
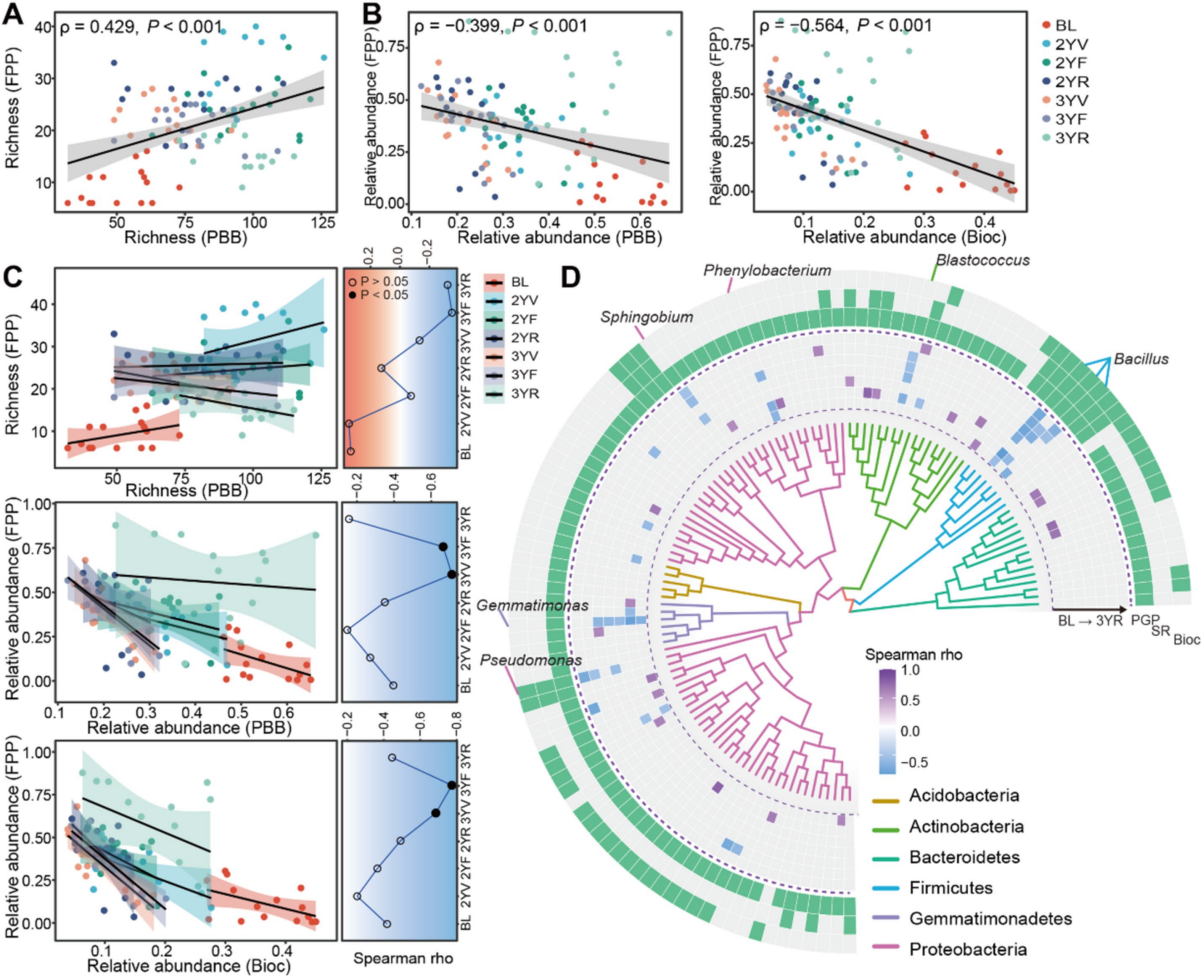
The potential antagonism effects between PBB and FPP and the succession patterns. **(A)** Correlations between the richness of PBB and FPP. **(B)** The correlation between the relative abundance of FPP and the relative abundance of PBB and Bioc communities, respectively. **(C)** Correlations between the indicators of PBB, Bioc, and FPP at different developmental stages. Line charts summarized the succession of correlation coefficients across plant developmental stages. Solid and hollow dots represented significant (*p* < 0.05) and non-significant (*p* > 0.05) correlations, respectively. **(D)** Significant correlations (BH adjusted *p* < 0.05) between widespread PBB ASVs (occurred in more than half of samples in different sampling stages) and the relative abundance of corresponding FPP community. The inside track represented phylogenetic tree of these bacterial ASVs constructed using FastTree. The heatmap in middle track represented the correlation coefficients. While the outside track indicated the functional subgroups of bacterial ASVs (i.e., PGP, SR, or Bioc). The genus-level taxonomic information was annotated for ASVs with negative and significant correlations with FPP across at least three sampling stages. PBB, FPP, PGP, Bioc, and SR represent plant-beneficial bacteria, fungal plant pathogens, plant-growth-promoting taxa, biocontrol taxa, and stress resistance provision taxa, respectively.

The relationships between PBB and FPP in terms of the biodiversity and relative abundance were further evaluated. A significant and positive correlation between the richness of PBB and FPP was observed (Spearman rank correlation, *ρ* = 0.429, *p* < 0.001), with the correlation coefficients varies at different growth stages (Figure 4A). However, the relative abundance of PBB was significantly and negatively correlated with that of FPP (*ρ* = 0.399, *p* < 0.001) (Figure 4B). Across different sampling stages, the PBB-FPP negative correlations varied, with the strongest negative correlations observed in the 3YV and 3YF stages (Figure 4C). In PBB subgroups, Bioc exhibited the strongest negative correlations with FPP (*ρ* = −0.564, *p* < 0.001) (Figures 4B,C), while the strengths of potential antagonism to FPP were lower for PGP (*ρ* = −0.391, *p* < 0.001) and SR (*ρ* = −0.465, *p* < 0.001) subgroups (Supplementary Figure S3).

The correlations between widespread ASVs and FPP were estimated for each development stage using spearman correlation analysis (Figure 4D). Bacterial taxa potentially antagonistic to FPP mainly belonged to Firmicutes, and exhibited the biocontrol function (Figure 4D). Only eight ASVs showed significant and negative correlations with FPP across at least three sampling stages, belonging to *Bacillus*, *Blastococcus*, *Phenylobacterium*, *Sphingobium*, *Pseudomonas*, and *Gemmatimonas* (Figure 4D).

**Figure 5:**
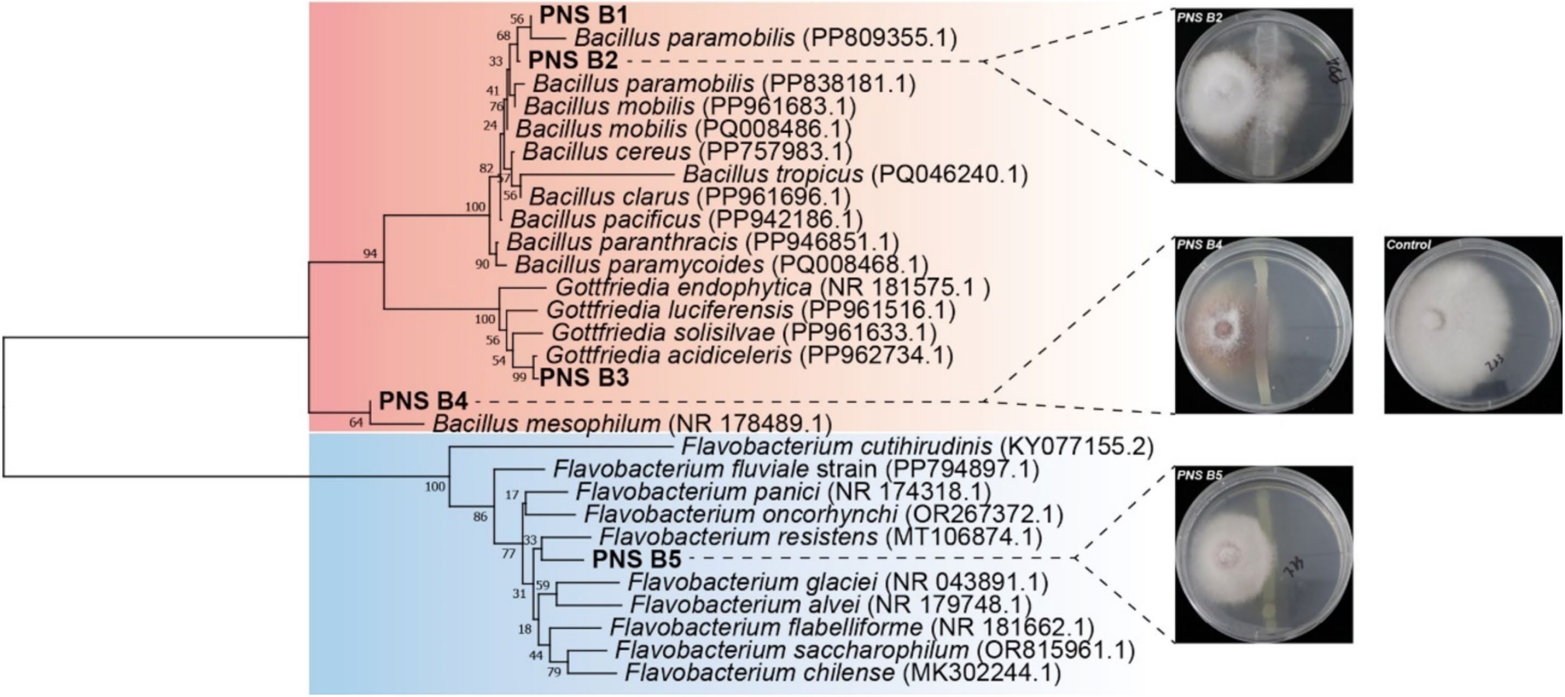
Evaluation of the role of Bioc’s antagonistic effect against *F. oxysporum*. Neighbor-joining tree showing the phylogenetic relationship among the isolated Bioc group strains obtained in this study (in bold). Accession numbers of isolates obtained from the NCBI database are displayed in regular letters. The middle pictures display the *in vitro* inhibitory effects of the three representative isolates on *F. oxysporum* using PDA assay. The picture on the right shows the control *F. oxysporum* culture medium without bacterial inoculation.

To confirm the antagonistic effect of the Bioc group against fungal pathogens in the rhizosphere of *P. notoginseng*, bacteria were isolated from the rhizosphere soil of *P. notoginseng*. According to the PBB database, five strains potentially exhibiting biocontrol capability were isolated, one belonging to *Flavobacterium*, three to *Bacillus*, and one to *Gottfriedia* (a new taxonomic group separated from *Bacillus*) (Figure 5). In which, the three representative isolates all could inhibit the growth of root rot pathogen *F. oxysporum* as indicated by the antagonism test, confirming the antagonistic effect of Bioc group in *P. notoginseng* rhizosphere (Figure 5). These results showed that potential antagonism effects exhibited between FPP and PBB especially the Bioc group, and the antagonism strength varied with plant growth. Simultaneously, the antagonistic experiment also verified the reliability of screening rhizosphere functional bacteria based on PBB database.

### 3.5 The relative contribution of deterministic and stochastic processes to the assembly of functional microbiomes varied with plant growth

**Figure 6:**
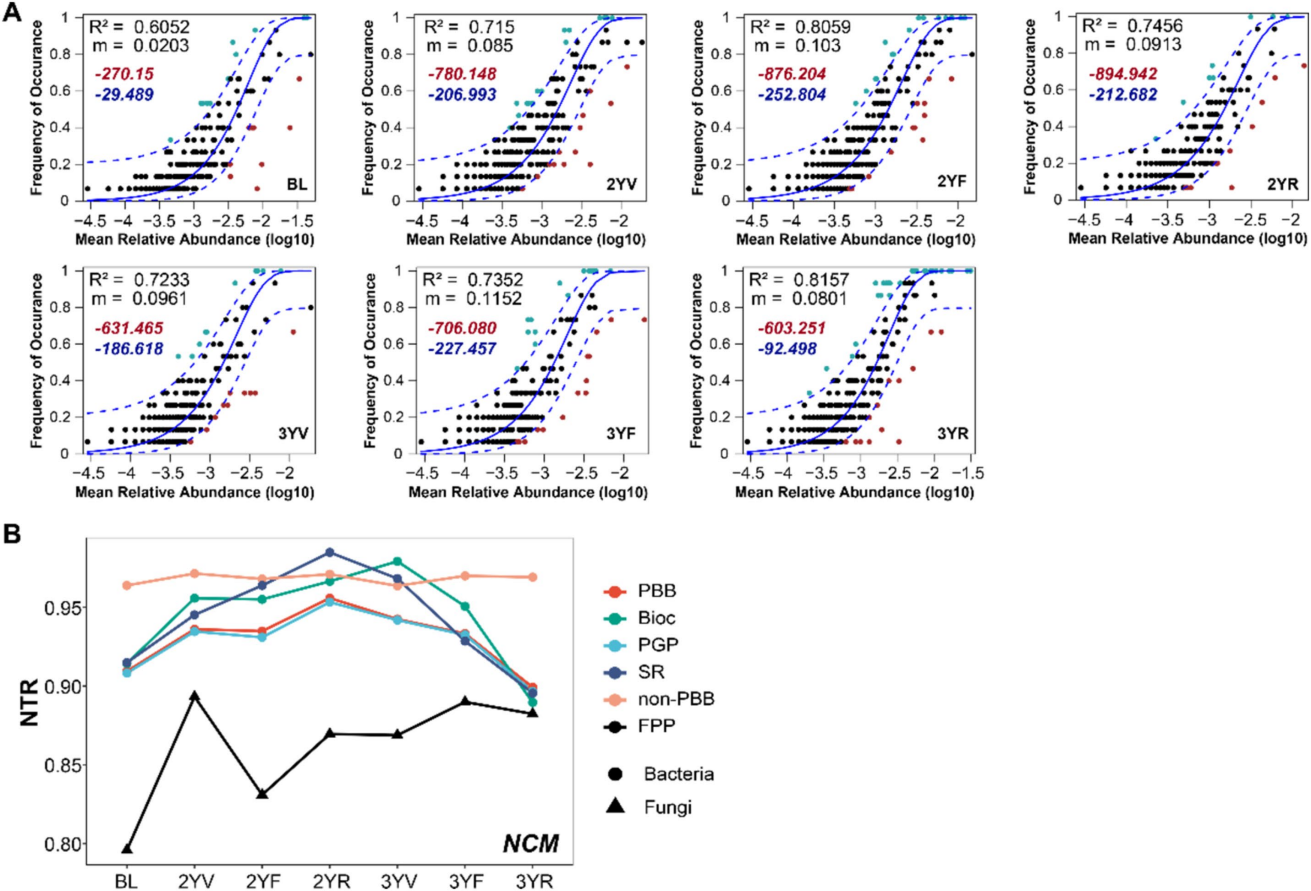
The ecological assembly mechanisms of rhizosphere functional microbiomes at different developmental stages of *P. notoginseng*. **(A)** Fit of the neutral community model with the community assembly of PBB. The solid blue line represents the best-fitting neutral model. The dashed line represents the 95% confidence intervals (CIs) around the best-fitting neutral model. m indicates the estimated migration rate, and R^2^ indicates the fit to the neutral model. Red and blue numbers denote the AIC of the neutral model and the binomial model, respectively. **(B)** The neutral taxa ratio (NTR) for the functional community. PBB, plant-beneficial bacteria; non-PBB, bacteria without plant-beneficial characteristics; FPP, fungal plant pathogens; PGP, plant-growth-promoting taxa; BIOC, biocontrol taxa; SR, stress resistance provision taxa.

The clear succession of PBB and FPP in the rhizosphere soil of *P. notoginseng* were driven by the underlying dynamics of ecological assembly processes. The occurrence and abundance of ASVs in different functional microbiomes sampled at distinct developmental stages were thus fitted using Sloan’s neutral model to evaluate the community assembly mechanisms (Figure 6; Supplementary Figures S4–7). The neutral model fitted well for FPP, PBB, and PBB subgroups, indicating the important role of stochastic processes to the assembly of functional microbiomes. AIC of neutral models were generally lower compared to corresponding binomial models, emphasizing the importance of dispersal limitation in shaping community structure (Figure 6A; Supplementary Figures S4–7).

The neutral taxa ratio was calculated to quantify the relative contributions of neutral (stochastic) and non-neutral (deterministic) processes to community assembly (Figure 6B). Compared with BL, the relative contribution of neutral process to the assembly of functional microbiomes showed a trend of first increasing and then decreasing. From the 3YV stage to the 3YR stage, the effects of deterministic processes increased for PBB and its subgroup communities, and the impact on FPP communities slightly increased. The null model-based NST analysis also confirmed the strong effects of deterministic processes at the 3YR period (Supplementary Figure S8). Notably, the NTR of non-PBB bacterial subcommunities did not change obviously across the growth period (Figure 6B). These results demonstrated that the succession of community assembly forces of functional microbiomes during the development stages of *P. notoginseng*.

### 3.6 Linking plant genetic network to the assembly of Bioc communities across plant development stages

**Figure 7:**
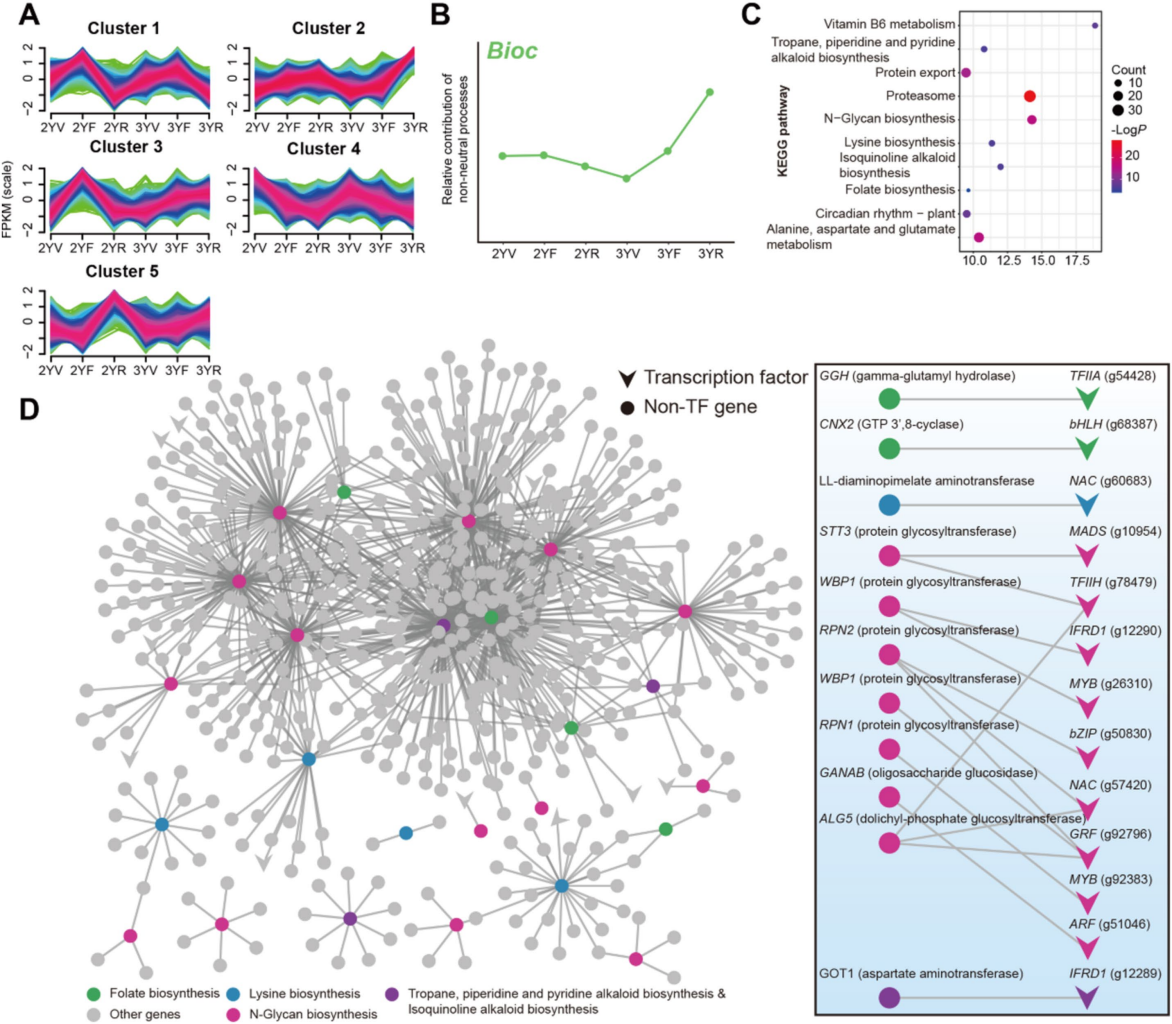
Gene expression analysis of *P. notoginseng* root at different developmental stages. **(A)** K-means-based gene clusters calculated using gene FPKM value. **(B)** The relative contribution of non-neutral processes to Bioc community assembly. **(C)** KEGG enrichment analysis of Clusters 2. Node size represents the number of genes within the pathway, and color represents the statistical significance. **(D)** Partial co-expression network of genes in cluster 2. Triangle and round nodes represented transcription factor (TF) and non-TF genes. The binary network on the right displayed the direct connections between TF and genes involved in enriched biosynthesis pathways. Gene symbols were labeled for biosynthesis-related genes, while TF families and annotation numbers were labeled for TFs.

As mentioned above, the Bioc taxa exhibited the strongest negative correlations with FPP, implying their potential in controlling fungal diseases. The links between plant root activities and the community assembly mechanisms of Bioc were thus investigated during plant growth. For the description of root activities, we collected root samples of *P. notoginseng* and performed RNA-seq analysis. In total, the expression of 39,238 genes in roots were quantified. K-means clustering analysis was performed on genes with annotated information, which were divided into five categories (Figure 7A). Cluster 2 showed a consistent trend with the relative contribution of deterministic processes on Bioc community, suggesting that these genes may be involved in the recruitment of Bioc community (Figure 7B). The KEGG pathway enrichment analysis results showed that cluster 2 was significantly enriched by functions related to the biosynthesis, metabolism, and exudation of metabolites in plants, including tropane, piperidine and pyridine alkaloid biosynthesis, N-glycan biosynthesis, lysine biosynthesis, isoquinoline alkaloid biosynthesis, folate biosynthesis, vitamin B6 metabolism, alanine, aspartate and glutamate metabolism and protein export (Figure 7C; Supplementary Table S1).

A co-expression network was then constructed for genes in cluster 2. Transcripts involved with the enriched biosynthesis pathways were extracted and visualized (Figure 7D). Genes related to the N-glycan biosynthesis and folate biosynthesis exhibited more connections compared to other functional genes (Figure 7D), indicating that their expression might be modulated by complex processes. Given the important role of transcription-level regulation in the expression of biosynthesis genes, the transcription factors (TF) connected to these genes were further extracted (Figure 7D). Thirteen TFs belonging to bHLH (g68387), NAC (g60683, g57420), MADS (g10954), MYB (g26310, g92383), bZIP (g50830), ARF (g51046), IFRD1 (g12290, g12289), GRF (g92796), TFIIA (g54428), and TFIIH (g78479) families were identified as potential regulators of biosynthesis pathways that might be related to the recruitment of Bioc taxa. These results revealed plant functions and regulators potentially related to the community assembly succession of rhizosphere Bioc community.

## 4 Discussion

Although extensive research has been conducted on rhizosphere functional microorganisms—both beneficial and harmful to the host plant—in terms of functional mechanisms, plant-microorganism interplays, and biogeographic distribution (Haskett et al., 2021; Li et al., 2023; Peng et al., 2023), their succession patterns, community assembly mechanisms, and links to plant genetic networks during the life cycle of perennial plants remain largely unexplored, which are critical for utilizing these functional microorganisms for sustainable agriculture. Dramatic physiological changes occur during plant development, such as resource allocation across organs, specific metabolite biosynthesis, and the balance between growth and defense (Chaparro et al., 2014; Major et al., 2017; Mathieu et al., 2009). These physiological variations, in conjunction with changing environmental conditions and ecological stochasticity, have significant impacts on rhizosphere functional microbiomes. Therefore, longitudinal studies encompassing different plant developmental stages, rather than merely cross-sectional analyses, are vital for a comprehensive understanding of plant-functional microorganism interactions and the effective utilization of microbes in sustainable agriculture (Russ et al., 2023; Xun et al., 2024). In the present study, the succession patterns of rhizosphere PBB and FPP in terms of biodiversity, relative abundance, and community structure across the life cycle of *P. notoginseng*, as well as the underlying assembly mechanisms were explored. The potential antagonism between PBB and FPP, as well as the links between the variations in root transcriptome and the deterministic assembly of beneficial microbial subcommunities were further investigated.

### 4.1 The succession patterns of rhizosphere functional microbiomes reflect the dynamic effects of host development

The biodiversity of both PBB and FPP in the rhizosphere of *P. notoginseng* was significantly higher than in unplanted bulk soil, aligning with findings from studies on other species like *Ranunculus glacialis* (Philippot et al., 2013). This increase can likely be attributed to the rhizodeposit effects of the host plant (Philippot et al., 2013). Rhizodeposits, including low-molecular-mass compounds exuded by roots, polymerized sugars, and root border cells, create diverse niches for rhizosphere functional microbes, thereby promoting species coexistence (Kraft et al., 2014). The niche-based promotion of species coexistence seems to be nonspecific to either PBB or FPP, as evidenced by significant and positive correlations between their *α*-diversities.

The biodiversity and relative abundance of PBB in the rhizosphere of *P. notoginseng* decreased and then increased throughout the plant’s development, reflecting the dynamic influence of the plant on its beneficial microbial inhabitants. An enhancement of rhizosphere effects with plant growth, similar to that observed in species like foxtail millet (Wang et al., 2022), suggests that plant requirements for and effects on beneficial bacteria might be minimal during the early slow growth stages (the 2nd year) but increase significantly in the 3rd year, especially during the root inflation stage (3YR). The specific rise in the relative abundance of PBB at 3YR may indicate plant recruitment of beneficial bacteria for essential functions such as nutrient uptake. For example, a notable increase in the relative abundance of Gemmatimonadetes at 3YR suggests their potential role in enhancing nitrogen supply to the host plant, as supported by metagenome-based studies (Kalam et al., 2022). This theory is further corroborated by neutral model findings, which shows a stronger relative contribution of selection processes to the assembly of PBB at the 3YR stage compared to earlier stages, a pattern not observed for non-PBB subcommunities. Similarly, both the relative abundance and deterministic assembly of FPP increased at 3YR, indicating that active root physiology at this stage attracts not only beneficial bacteria but also potentially pathogenic fungi. Prior research has highlighted the crucial roles of root exudates in plant defense against pathogens, such as coumarins and benzoxazinoids (Pascale et al., 2019). However, the specifics of which root-derived molecules attract plant pathogens remain largely unexplored and warrant further investigation (van Griethuysen et al., 2024).

Another critical period is the 3YV stage, during which the relative abundance of both functional microbiomes, including PBB and FPP, were at their lowest. During the winter between the 2YR and 3YV stages, the reduced physiological activities of the plant, due to leaf abscission and root dormancy, likely lead to diminished effects on the functional microbiomes. Consequently, these microorganisms, heavily reliant on plant-derived carbon resources, might struggle to compete with other microbial taxa in the absence of plant support (Russ et al., 2023). This observation underscores the significance of continuous carbon input from the plant in maintaining rhizosphere functional microbiomes.

### 4.2 The potential antagonism in the rhizosphere reduces at the rapid growth stage of host plant

This study’s findings align with our hypothesis, revealing significant and negative correlations between the relative abundance of FPP and PBB, particularly within the Bioc subgroup. The finding was also supported by the isolation-based antagonism test experiments. Biocontrol bacteria are known to suppress FPP proliferation through various mechanisms, including host immune induction, antibiosis compound production, and competition for scarce resources (Kohl et al., 2019). These complex interactions likely contribute to the strong negative correlations observed in our study. *Bacillus* spp. and *Pseudomonas* spp. are bacteria in the Bioc subgroup that show significant and negative correlations with FPP. Both genera have been well studied and their abilities to protect plants against pathogens have been widely described (Bernal et al., 2017; Molina-Santiago et al., 2019). The Bioc subgroup identified here presents a promising avenue for microbe isolation and validation for agricultural applications.

A strong succession patterns of the potential antagonism interactions were also observed during *P. notoginseng* growth. The potential antagonism continuously strengthened from 2YV to 3YV, as indicated by the negative correlation coefficients, implying the increasing regulation of plant on Bioc bacteria for protection against pathogens. However, at the 3YR stage, there was a marked weakening of this antagonistic strength. Previous research has focused on the “growth-defense trade-offs,” particularly in terms of genetic regulatory networks in plants involving diverse hormones, pathogen pattern recognition, and immune responses (He et al., 2022; Major et al., 2017). Our finding of diminished rhizosphere antagonism during rapid plant growth suggests that microbe-dependent rhizosphere defense might be an overlooked aspect of this trade-off. During periods of accelerated root biomass accumulation, plant physiological activities might prioritize primary metabolism and nutrient uptake, potentially at the expense of root defense, including microbe-assisted pathogen antagonism. Another possibility is that plant roots release more resources at the 3YR stage, weakening the resource competition-based antagonism in the rhizosphere. This insight underscores the importance of monitoring rhizosphere antagonistic interactions within agricultural ecosystems, especially during critical growth phases.

### 4.3 Plant genetic networks potentially related to the deterministic assembly of rhizosphere Bioc bacterial subcommunity

Genetically determined rhizodeposits are crucial for shaping the structure and function of the rhizosphere microbial community (Philippot et al., 2013). Thus, understanding the genetic basis of beneficial bacterial community dynamics is essential for breeding *P. notoginseng* varieties that are optimized for rhizosphere microbiome assistance (Trivedi et al., 2020; Xun et al., 2024). The exudation of proteins might be involved in important signaling molecules regulating the interaction between plants and microorganism, affecting the formation of beneficial rhizosphere microbial communities (Galloway et al., 2020; Sasse et al., 2018). N-glycan is an important protein modification (Ruiz-May et al., 2012), and our research has determined that the functional pathway of N-glycan biosynthesis in *P. notoginseng* is related to the deterministic assembly of Bioc community. The biosynthesis of other compounds, including different types of alkaloids, amino acid, and folate, were also linked to the assembly of rhizosphere Bioc community. The root exudation of these compounds has been extensively reported (Afridi et al., 2024; Chaparro et al., 2014). These complex groups of compounds can collectively shape beneficial rhizosphere microbiomes through multiple mechanisms, including chemotaxis and microenvironment regulation (Afridi et al., 2024). More importantly, transcription factors belonging to various families including MADS, TFII, IFRD1, MYB, bZIP, NAC, GRF and ARF, were identified as potential regulators controlling the biosynthesis of these compounds, potentially regulating the assembly and succession of FRM communities. For example, transcription factors MYB72 and MYB72-controlled *β*-glucosidase BGLU42 play a key role in regulating beneficial rhizosphere bacteria-induced systemic resistance (ISR) and by modulating the secretion of coumarin to inhibit soil-borne fungal pathogens (Stringlis et al., 2018). The plant functional pathways and regulators identified in this study offer valuable insights into the molecular mechanisms that underlie the interactions between plants and beneficial microbes in the rhizosphere, and provide clues for further molecular verification.

### 4.4 Fully understanding plant-rhizosphere interactions in the agricultural system of perennial plants: future directions

Although this study revealed the rhizosphere functional microorganisms, PBB-FPP interactions, the succession of beneficial microbial ecological construction processes during *P. notoginseng* growth, as well as the related plant genetic factors, more efforts are still needed to fully understand the interactions between plants and rhizosphere in perennial plant agricultural systems, in order to achieve microbial-based sustainable agricultural practices. Firstly, the candidate plant gene list was obtained through correlation analysis. Further validation studies using genetic manipulation techniques such as gene editing will confirm the functions of these candidate genes, providing targets for plant breeding aimed at healthy rhizosphere microbial communities. Secondly, although sampling was conducted at various developmental stages of *P. notoginseng* in this study, the rhizosphere exhibited complex dynamic changes, and the sampling frequency may not be sufficient to capture the complete dynamic patterns. Hence, more intensive sampling of both plants and the rhizosphere should be included in future studies. Third, the dynamics of the rhizosphere microbiome throughout the entire growth cycle of model plants and crops (such as *Arabidopsis*, rice, etc.) (Chaparro et al., 2014; Zhang et al., 2018) had been extensively studied, but the study of the succession patterns of the rhizosphere microbial communities during the developmental stages of perennial plants is relatively scarce. In future studies, more well-designed plant-rhizosphere microbe interaction studies on perennial plants are needed to help improve agricultural practices for perennials.

## 5 Conclusion

In conclusion, the plant developmental stage was a significant driving force affecting the rhizosphere functional microbiomes. The relative impact of deterministic processes on the assembly of functional microbial communities showed a trend of first increasing and then decreasing during plant growth, reflecting the adaptability of plant functional microbial communities to plant developmental changes. The genetic regulation networks, functional pathways, and potential molecular regulators related to the deterministic assembly of biocontrol rhizosphere microbiomes were also identified. This work provides a comprehensive understanding of the assembly and succession of rhizosphere functional microbiomes, as well as their links to plant genetic networks across the life cycle of perennial *P. notoginseng*, which is vital for utilizing microbial resources to achieve sustainable agriculture of medicinal plant.

## Data Availability

The original contributions presented in the study are included in the article/Supplementary material, further inquiries can be directed to the corresponding authors.

## Footnotes

^1^Plant-beneficial bacteria

^2^Biocontrol taxa

^3^Plant-growth-promoting taxa

^4^Stress resistance provision taxa

^5^Fungal plant pathogens

^6^ http://www.r-project.org/
