# Supplementary material for: Understanding the influence of plant genetic factors on rhizosphere microbiome assembly in Panax notoginseng
Source: Front Microbiol. 2024 Dec 16;15:1479580. doi: 10.3389/fmicb.2024.1479580 (PMC11683141; doi:10.3389/fmicb.2024.1479580)
Supplement: Supplementary file 2 [file Image_1.pdf]

# Linking plant genetic regulatory network to the assembly and succession of rhizosphere functional microbiomes across the life cycle of perennial *Panax notoginseng*

Liping Shi[1a], Mingming Yang[1a], Guangfei Wei[1], Xiuye Wei[1, 3], Fei Hong[2], Jiaxiang Ma[1], Zhe Wu[1], Yuqing Zheng[2], Miyi Yang[1], Shilin Chen[1,3], Guozhuang Zhang[1**], Linlin Dong[1*]

[1] *State Key Laboratory for Quality Ensurance and Sustainable Use of Dao-di Herbs, Institute of Chinese Materia Medica, China Academy of Chinese Medical Sciences, Beijing, 100700, China*

[2] *Zhangzhou Pien Tze Huang Pharmaceutical Co., Ltd., Fujian, 363000, China*

[3] *Institute of Herbgenomics, Chengdu University of Traditional Chinese Medicine, Chengdu, Sichuan, China*

**E-mail address:**

Liping Shi: shiliping10@126.com

Yang mingming: yangmingming2013@163.com

Guangfei Wei: gfwei@icmm.ac.cn

Xiuye Wei: xiuyewei1220@126.com

Fei Hong: pzhhf123@126.com

Jiaxiang Ma: 2065463521@qq.com

Zhe Wu: zwu@icmm.ac.cn

Yuqing Zheng: zyq@zzpzh.com

Miyi Yang: myyang@icmm.ac.cn

Shilin Chen: slchen@icmm.ac.cn

[a] These authors contributed equally to this work.

** **Corresponding author**

TEL: (+86) 18811719589; email: gzzhang@icmm.ac.cn

* **Corresponding author**

TEL: (+86) 18911917789; email: lldong@icmm.ac.cn

*State Key Laboratory for Quality Ensurance and Sustainable Use of Dao-di Herbs, Institute of Chinese Materia Medica, China Academy of Chinese Medical Sciences, Beijing, 100700, China*

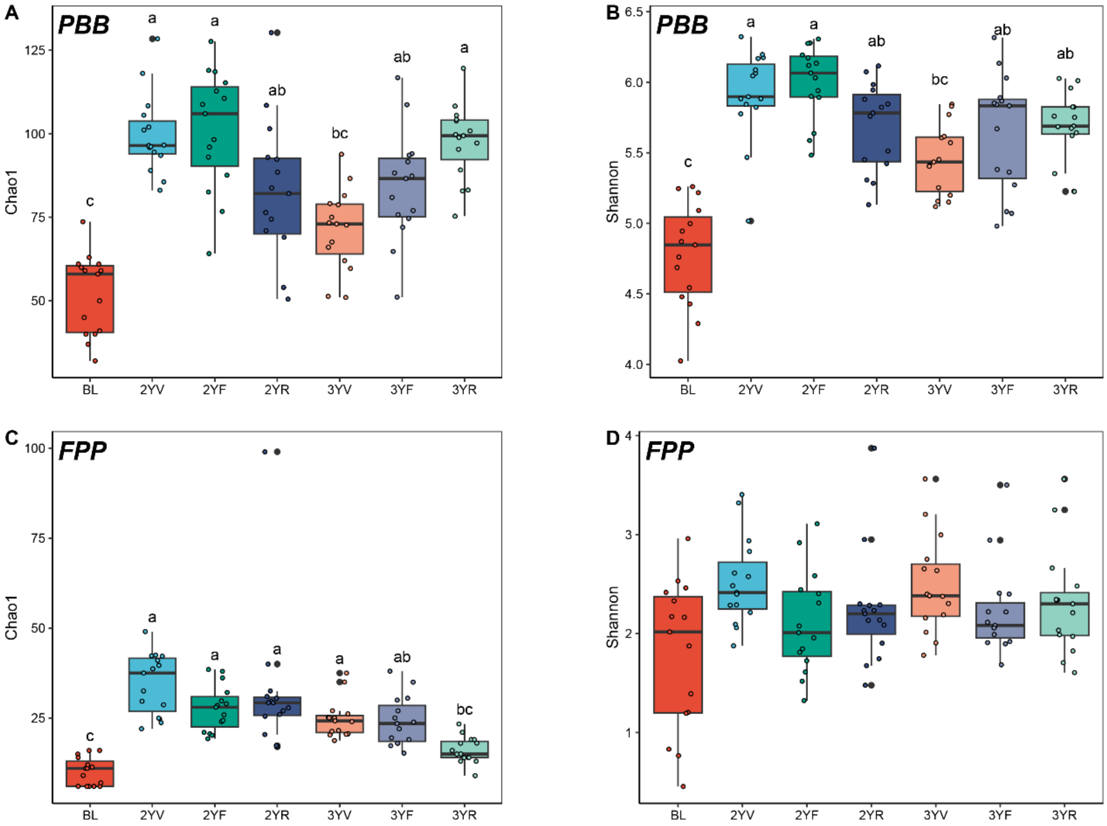


**Figure S1. Changes in α-diversities of PBB and FPP communities with *P. notoginseng* growth.** PBB α-diversities at different growth stages are represented by ASV Chao1 **(A)** and Shannon **(B)** index. FPP α-diversities at different growth stages are represented by ASV Chao1 **(C)** and Shannon **(D)** index. BL, 2YV, 2YF, 2YR, 3YV, 3YF, and 3YR represent BLs and the 2-year vegetative, 2-year flowering, 2-year root growth, 3-year vegetative, 3-year flowering, and 3-year root growth stages, respectively. Different letters above the boxes indicate a significant difference determined by nonparametric Kruskal Wallis test ($P < 0.05$).

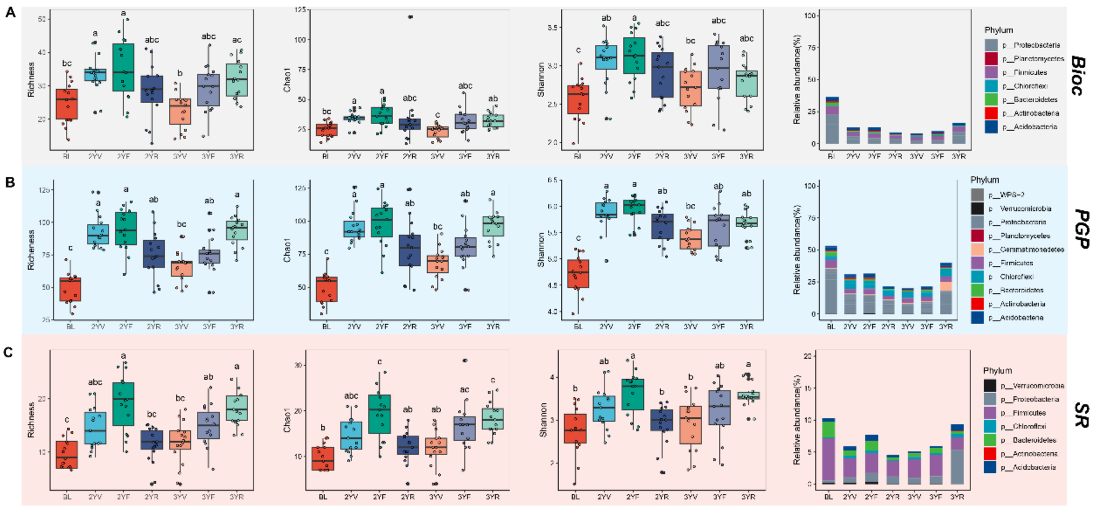


**Figure S2. Changes in α-diversities and composition of Bioc (A), PGP (B), and SR (C) communities with *P. notoginseng* growth. Different letters above the boxes indicate a significant difference determined by Kruskal-Wallis followed by Nemenyi test ($P < 0.05$).**

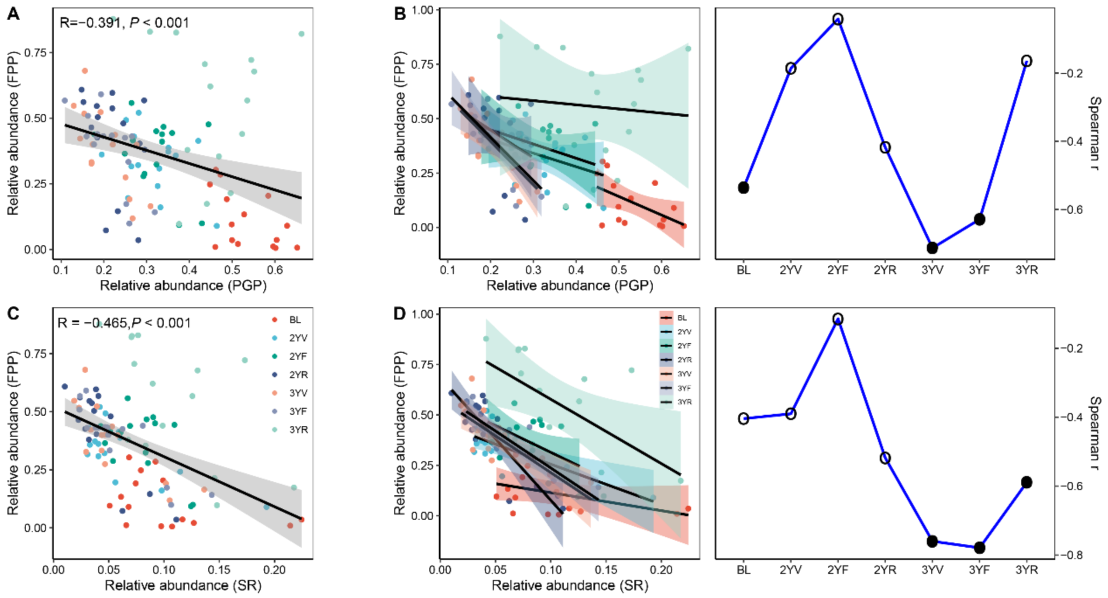


**Figure S3. The relationship between the biodiversity and relative abundance of PBB subgroups and FPP, and the succession pattern of the correlation during the growth stages of *P. notoginseng*.** **(A)** Correlations between the relative abundance of PGP and FPP. **(B)** Correlations between the relative abundance of PGP and FPP at different developmental stages. **(C)** Correlations between the relative abundance of SR and FPP. **(D)** Correlations between the relative abundance of SR and FPP at different developmental stages.

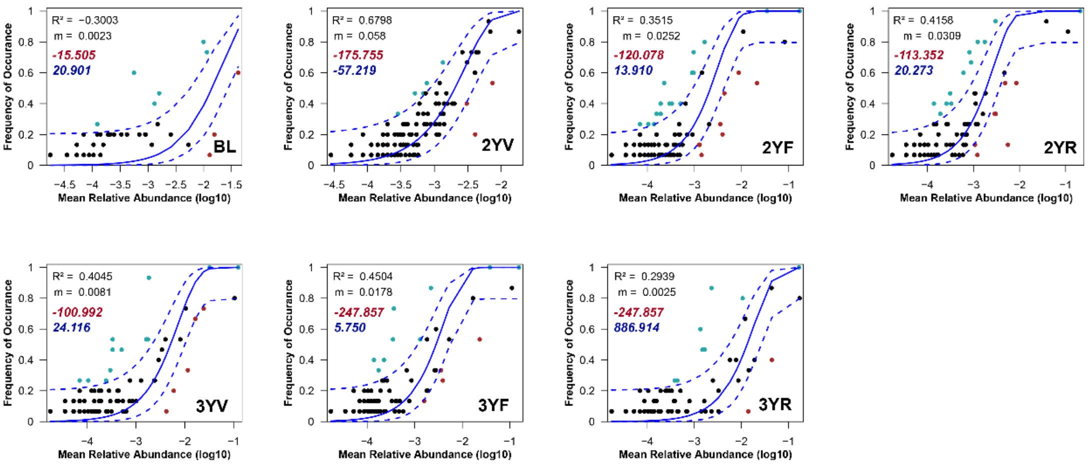


***FPP***

**Figure S4. Fit of the neutral community model with the community assembly of FPP.** The solid blue line represents the best-fitting neutral model. The dashed line represents the 95% confidence intervals (CIs) around the best-fitting neutral model. ASVs within the CIs (black points) follow the neutral process. ASVs that occur more frequently than predicted by the model are shown in blue, whereas those that occur less frequently than predicted are shown in red. m indicates the estimated migration rate, and $R^2$ indicates the fit to the neutral model. Red and blue numbers denote the AIC of the neutral model and the binomial model, respectively.

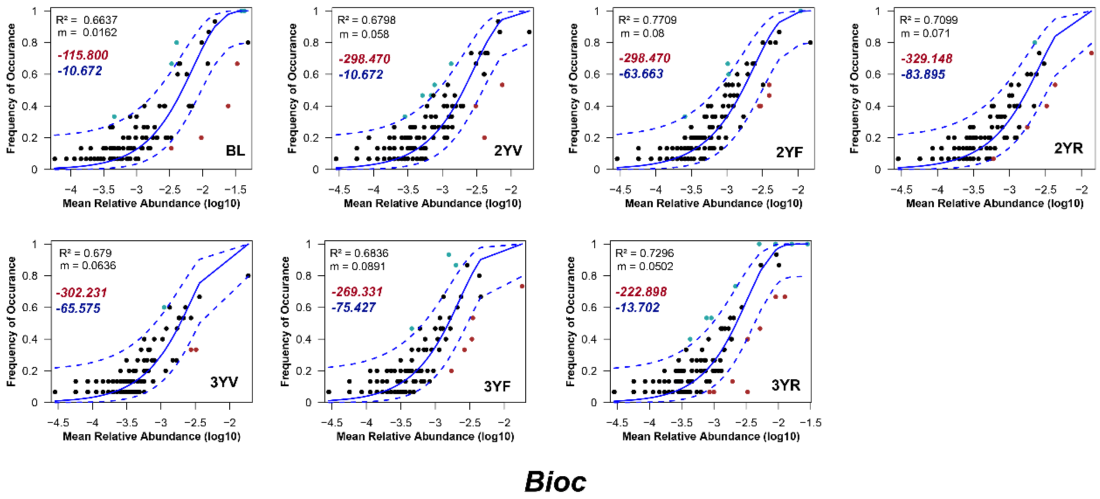


**Figure S5. Fit of the neutral community model with the community assembly of Bioc.**

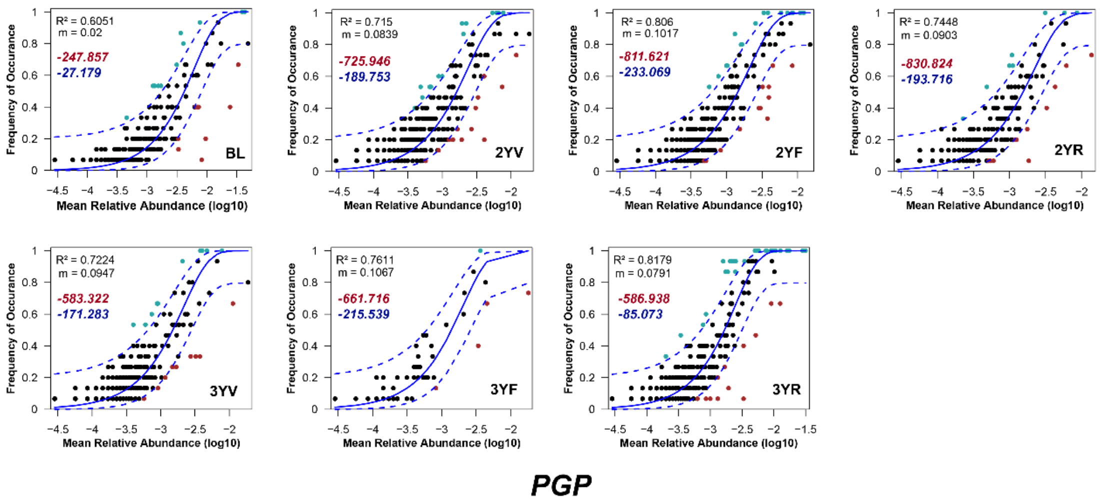


**Figure S6. Fit of the neutral community model with the community assembly of PGP.**

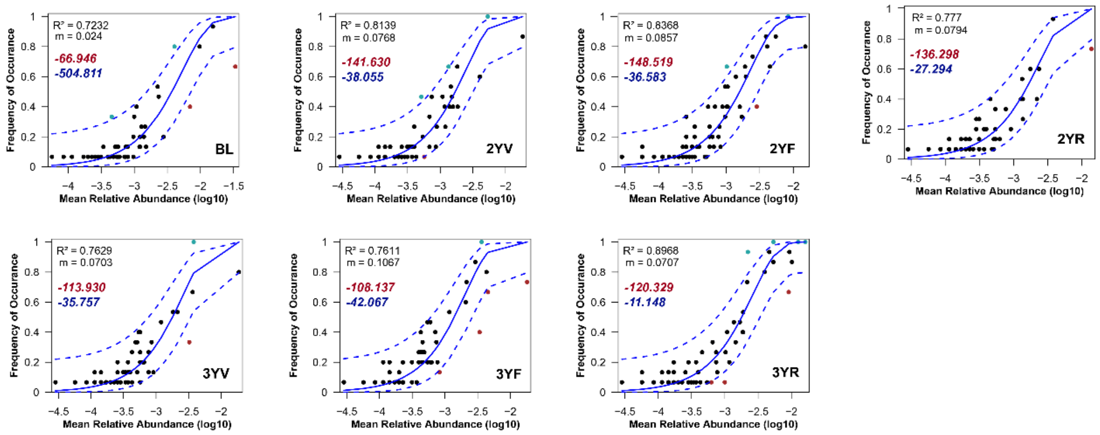


***SR***

**Figure S7. Fit of the neutral community model with the community assembly of SR.**

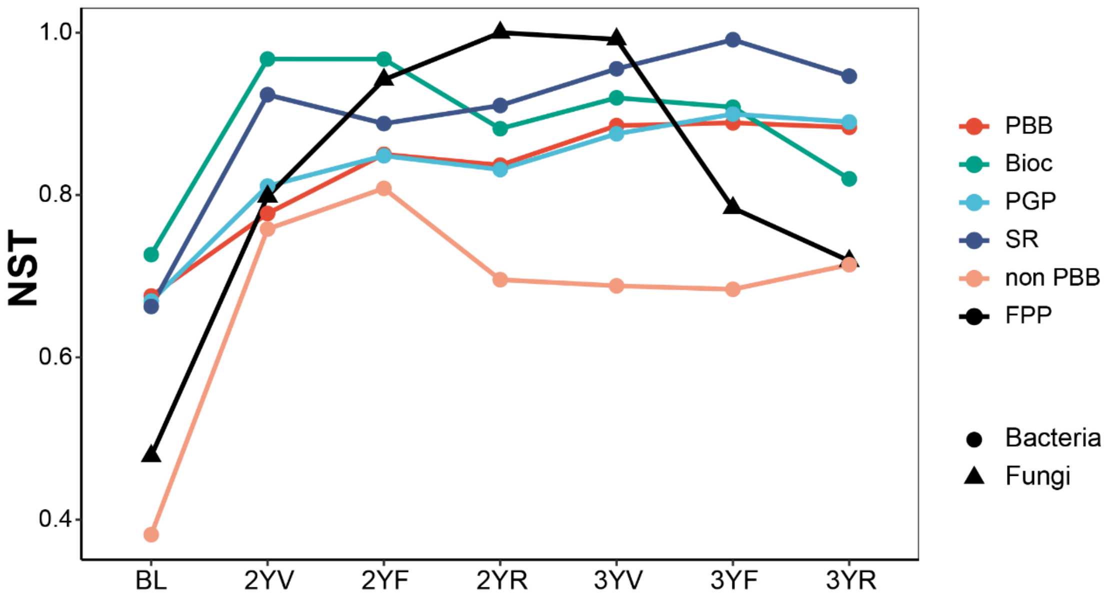


**Figure S8. Normalized stochasticity ratio (NST) estimated stochasticity in functional community assembly.** Bacteria, all bacterial functional groups and non-PPB; Fungi, fungal plant pathogens.
